# Co-Precipitation, Strength and Electrical Resistivity of Cu–26 wt % Ag–0.1 wt % Fe Alloy

**DOI:** 10.3390/ma10121383

**Published:** 2017-12-03

**Authors:** Rui Li, Engang Wang, Xiaowei Zuo

**Affiliations:** 1 Key Laboratory of Electromagnetic Processing of Materials (Ministry of Education), Northeastern University, Shenyang 110819, China; neulirui@hotmail.com; 2 School of Materials Science and Engineering, Northeastern University, Shenyang 110004, China; 3 School of Metallurgy, Northeastern University, Shenyang 110004, China

**Keywords:** Cu–Ag alloy, Fe addition, co-precipitation, precipitation kinetics, hardness, electrical resistivity

## Abstract

Both a Cu–26 wt % Ag (Fe-free) alloy and Cu–26 wt % Ag–0.1 wt % Fe (Fe-doping) alloy were subjected to different heat treatments. We studied the precipitation kinetics of Ag and Cu, microstructure evolution, magnetization, hardness, strength, and electrical resistivity of the two alloys. Fe addition was incapable of changing the precipitation kinetics of Ag and Cu; however, it decreased the size and spacing of rod-shaped Ag precipitates within a Cu matrix, because Fe might affect the elastic strain field and diffusion field, suppressing the nucleation of Ag precipitates. Magnetization curves showed that γ-Fe precipitates were precipitated out of the Cu matrix, along with Ag precipitates in Fe-doping alloy after heat treatments. The yield strength of the Fe-doping alloy was higher than that of the Fe-free alloy, and the maximum increment was about 41.3%. The electrical conductivity in the aged Fe-doping alloy was up to about 67% IACS (International Annealed Copper Standard). Hardness, strength, and electrical resistivity were intensively discussed, based on the microstructural characterization and solute contributions of both alloys. Our results demonstrated that an increasing fraction of nanoscale γ-Fe precipitates and decreasing spacing between Ag precipitates resulted in the increasing strength of the Fe-doping alloy.

## 1. Introduction

High-strength, high-conductivity materials are required extensively in the construction of high-field magnets [1,2]. Cu and Cu–X alloys (X = Ag, Fe, Nb, Cr, etc.) have attracted considerable attention as winding conductors, owing to their superior combinations of strength and electrical conductivity [3,4,5,6]. Cu–Ag alloys have been widely involved in all of these materials [7,8,9,10]. The microstructure of a Cu–Ag alloy with more than 6 wt % Ag is composed of a Cu matrix, embedded by Ag precipitates and eutectic colonies [11]. The strength of the Cu–Ag alloy can be increased by refining Cu dendrites and Ag precipitates [10]. Zuo et al. [12] investigated the contributions of individual microstructures to the strength and electrical conductivity of Cu–28 wt % Ag composite, and revealed that the spacing of Ag precipitates took a dominant role in the two properties [12]. Ageing treatment caused Ag to precipitate out of the Cu matrix, resulting in precipitation hardening [2]. Optimization of Ag precipitates in a Cu matrix has been investigated by adjusting ageing [13,14,15], exploring the thermos-mechanical process [16,17], adding third elements [18,19], etc. These ways were effective at improving the properties of Cu–Ag alloy.

This Cu–Fe alloy also serves as a good candidate for conductive materials, because of the low costs of iron compared to other insoluble elements [6,20,21], as well as its excellent mechanical properties [22,23]. According to the Cu–Fe phase diagram [24], the maximum solubility of iron in copper is 4.1 wt %, and its minimum solid solubility at room temperature is significantly lower than 1 wt %. It is possible to precipitate Fe out of a Cu matrix, and enhance strength and electrical conductivity [21,25,26]. However, the application of the Cu–Fe alloys has been limited, because serious composition segregation takes place during solidification [27]. The relatively high solubility of iron in copper at high temperatures and the slow kinetics of iron precipitation at low temperature [28,29] restrict the precipitation of Fe out of the Cu matrix, thus keeping electrical conductivity at a lower level.

Some studies have paid attention to Cu–Ag–Fe systems because of the higher elastic modulus (211 GPa) of Fe compared to Cu (129.8 GPa) and Ag (82.7 GPa) [30]. Additionally, the co-deformation between the face-centered-cubic phase (Cu, Ag) and body-centered-cubic phase (Fe) may increase interface density, so as to increase interface strengthening. The introduction of Ag into Cu–Fe alloy refined the primary Fe dendrites [31,32], improved Fe precipitation [33,34,35], and enhanced the alloy’s strength and electrical conductivity [36,37]. However, the effects of Fe addition on precipitation kinetics and morphology of Ag precipitates are rarely reported. In this work, we choose minor Fe of about 0.1 wt % as a third element to add into a Cu–26 wt % Ag alloy, and investigate the effect of Fe addition on precipitation kinetics and microstructures of Ag precipitates. The relationship between microstructures, strength, and resistivity in both alloys was also discussed. It is of scientific interest to clarify the interaction between co-precipitations, and a diagram of their performances will be drawn in order to find other potential applications such as transmission lines.

## 2. Experimental Procedure

Both Cu–26 wt % Ag (Fe-free) ingots and Cu–26 wt % Ag–0.1 wt % Fe (Fe-doping) ingots (actual chemical compositions) were prepared in a vacuum-induction melting furnace. The as-cast ingots were homogenized at 760 °C for 24 h, and subsequently quenched in water at room temperature. The as-solid-solution samples were sectioned into several rectangular specimens (length of 120 mm and thickness of 2 mm). The samples were aged at temperatures of 200–550 °C for 2–16 h, and cooled in a furnace under an argon atmosphere.

The aged samples were ground on SiC papers and polished, before examining scanning electron microscopy (SEM). SEM (ULTRA PLUS, Zeiss, Germany) was carried out using Zeiss field emission scanning electron microscopy (FESEM) operating at 20 kV. Thin foils for transmission electron microscopy (TEM) were prepared by grinding a 3-mm diameter disk to 30 μm in thickness, and ion-milling (PIPS II 695 Gatan, Pleasanton, CA, USA) with a voltage of 5 kV and a gun tilt angle of 5° at liquid nitrogen temperature. Morphology of precipitates was observed on an FEI-Tecnai G^2^ 20 TEM (FEI, Hillsboro, OR, USA) equipped with an energy-dispersive X-ray spectroscopy (EDS, FEI, Hillsboro, OR, USA) detector operating at 200 kV. Precipitation kinetics of the two as-solid-solution alloys were determined using differential scanning calorimetry (DSC, Netzsch–Proteus–61, NETZSCH (Shanghai) Machinery and Instruments Co., Ltd., Shanghai, China) with different heating rates (10–50 °C/min), from room temperature to 700 °C. Magnetic hysteresis loops were measured at room temperature, on the samples with 3 × 3 × 1 mm^3^. Hardness measurement was conducted with a load of 100 g and a dwelling time of 10 s. Tensile tests (AG-X 100 kN, Shimadzu, Chiyoda-ku, Tokyo, Japan) were carried out at room temperature, with an initial strain rate of 10^−4^ s^−1^.

## 3. Results

### 3.1. Precipitation Kinetics of Ag and Cu

**Figure 1:**
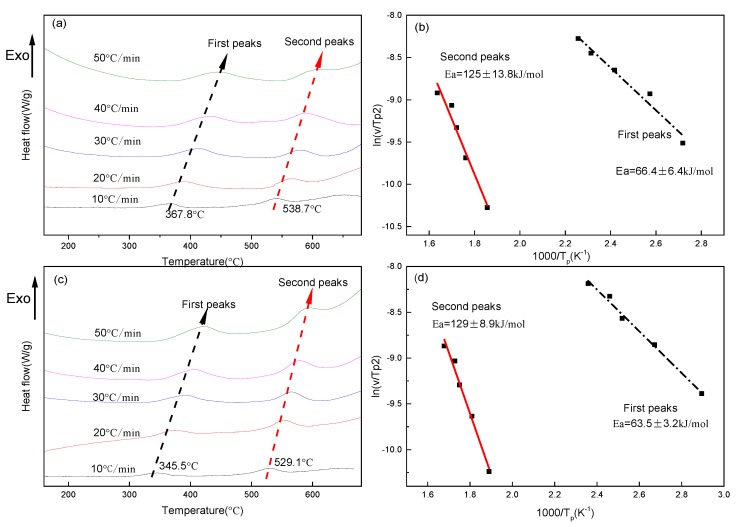
Differential scanning calorimetry (DSC) heating curves of as-solid-solution (**a**) Fe-free alloy and (**c**) Fe-doping alloy. (**b**,**d**) Show Kissinger plots for the activation energies of the reactions, from the first temperature peaks and the second temperature peaks.

DSC curves of the two as-solid-solution alloys show two exothermic reactions during heating up (Figure 1a,c). The temperature peaks of the curves shift toward higher temperatures with an increasing heating rate. Figure 1a shows that the first set of peaks is at temperatures between 367 °C and 425 °C, indicating the precipitation reaction of Ag out of the Cu matrix [13,38], and that the second set of peaks appears at temperatures between 538 °C and 600 °C, indicating the precipitation reaction of Cu out of the Ag matrix [39]. The comparison betweenFigure 1a and Figure 1c shows that the two sets of peak temperatures decreased by about 10–20 °C at each DSC curve after Fe-doping.

The activation energies (*E*_a_) of Ag precipitates and Cu precipitates at various heating rates were calculated according to the DSC curve by the Kissinger equation [40]:

(1) ln(vTp2)=−EaRTp+C

where *v* is the heating rate, *C* is a constant, *E*_a_ is the activation energy (kJ/mol), *T*_p_ is the temperature peak observed in the DSC curves, and R is the gas constant.

Equation (1) indicates that there is a linear relationship between ln (*v*/*T_p_*^2^) and (1000/*T_p_*). For the Fe-free alloy, the values of the activation energy of the first and the second set of peaks are estimated as 66.4 ± 6.4 kJ/mol and 125 ± 13.8 kJ/mol, respectively (Figure 1b), where the deviations resulted from the experimental errors of DSC facilities, weight measurement errors of the samples, and chemistry variations among the samples. The values of the activation energy of Ag precipitates out of the Cu matrix are consistent with those measured by DSC in Cu–28 wt % Ag alloy (55.3 ± 22.4 kJ/mol) [14], in Cu–8 wt % Ag alloy (63.7 ± 0.1 and 68.7 ± 2.3 kJ/mol) [13], and in Cu–7 wt % Ag alloy (95 ± 4.2 kJ/mol) [41]. The values of the activation energy of Cu precipitates out of the Ag matrix are comparable with those obtained by DSC in Ag–7.5 wt % Cu alloy (110 ± 8 kJ/mol) [42], and in Ag–8 wt % Cu alloy (109 ± 6.7 kJ/mol) [43]. For the Fe-doping alloy (Figure 1d), the values of the activation energy of the first and second set of peaks are estimated as 63.5 ± 3.2 kJ/mol and 129 ± 8.9 kJ/mol, respectively. Fe addition may slightly decrease the activation energies of Ag precipitates out of the Cu matrix, and marginally increase the activation energies of Cu precipitates out of the Ag matrix. The precipitation of Fe out of the Cu matrix in a Cu–4.4 wt % Fe alloy by DSC ranged from 450 °C to 600 °C [44]. In our Fe-doping alloy, because of the few Fe additions, the peak temperatures of exothermic heat flow from the precipitation of Fe out of the Cu matrix might overlap with the exothermic reactions from the precipitation of Cu out of the Ag matrix. It is hard to exclude the effect of Fe precipitation on the activation energies established from the second set of temperature peaks, which might be one of the reasons to marginally increase the activation energies by Fe-doping from 125 ± 13.8 kJ/mol to 129 ± 8.9 kJ/mol.

### 3.2. Morphology of Alloys

#### 3.2.1. Morphology of Eutectic Colonies

**Figure 2:**
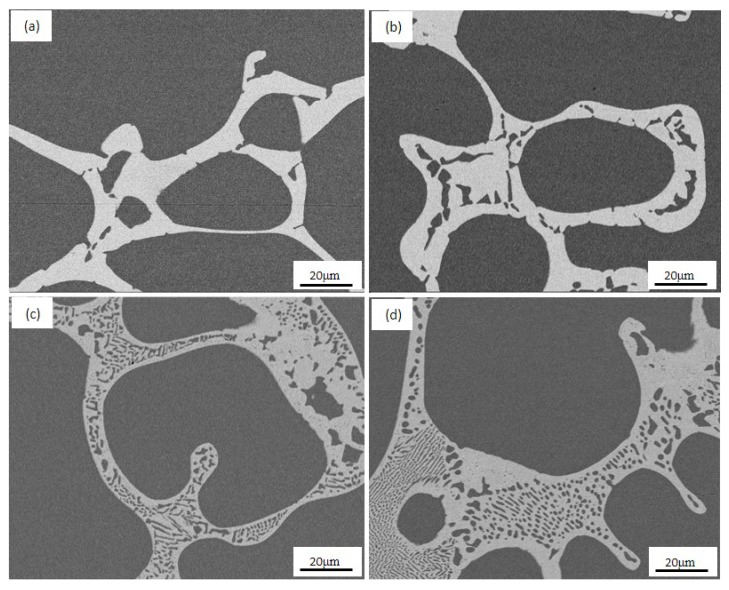
Microstructure of eutectics in the Fe-free alloy: (**a**) as-solid-solution, (**c**) 550 °C-4 h, and in the Fe-doping alloy: (**b**) as-solid-solution, (**d**) 550 °C-4 h.

Figure 2 shows typical microstructures of both Fe-free and Fe-doping alloys. The microstructure consists of two phases: proeutectic Cu (dark contrasts) and eutectic components (light contrasts). The volume fraction of eutectic in the as-solid-solution Fe-free alloy is about 17.4%, which is lower than that of 19.1% for the as-solid-solution Fe-doping alloy. After ageing treatment at 550 °C, the volume fraction of both alloys increases to 25% and 26.2%, respectively.

#### 3.2.2. Morphology of Ag Precipitates in the Cu Matrix

**Figure 3:**
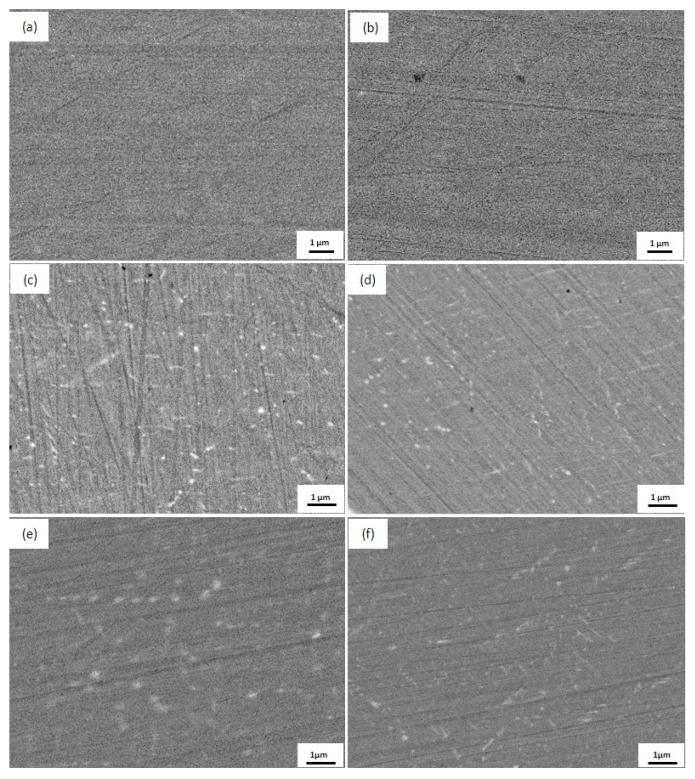
Microstructure of continuous Ag precipitates in the Cu matrix of solution-aged samples at various temperatures for 4 h. Fe-free alloy: (**a**) as-solid-solution, (**c**) 450 °C, (**e**) 500 °C, and Fe-doping alloy: (**b**) as-solid-solution, (**d**) 450 °C, and (**f**) 500 °C.

No Ag precipitate was found in the Cu matrix after water quenching by SEM (Figure 3a,b). Ageing at 450 °C caused continuous Ag precipitates to nucleate in both alloys, and large-scale Ag precipitates, with a diameter of 40–50 nm, dispersed uniformly in the Cu matrix (Figure 3c,d). After increasing the temperature to 500 °C (Figure 3e,f), the Ag precipitates in the two alloys grew slightly larger than those at 450 °C. The size of the Ag precipitates in the Fe-doping alloy was smaller compared with the Fe-free alloy, which can be seen in Figure 3b–f.

**Figure 4:**
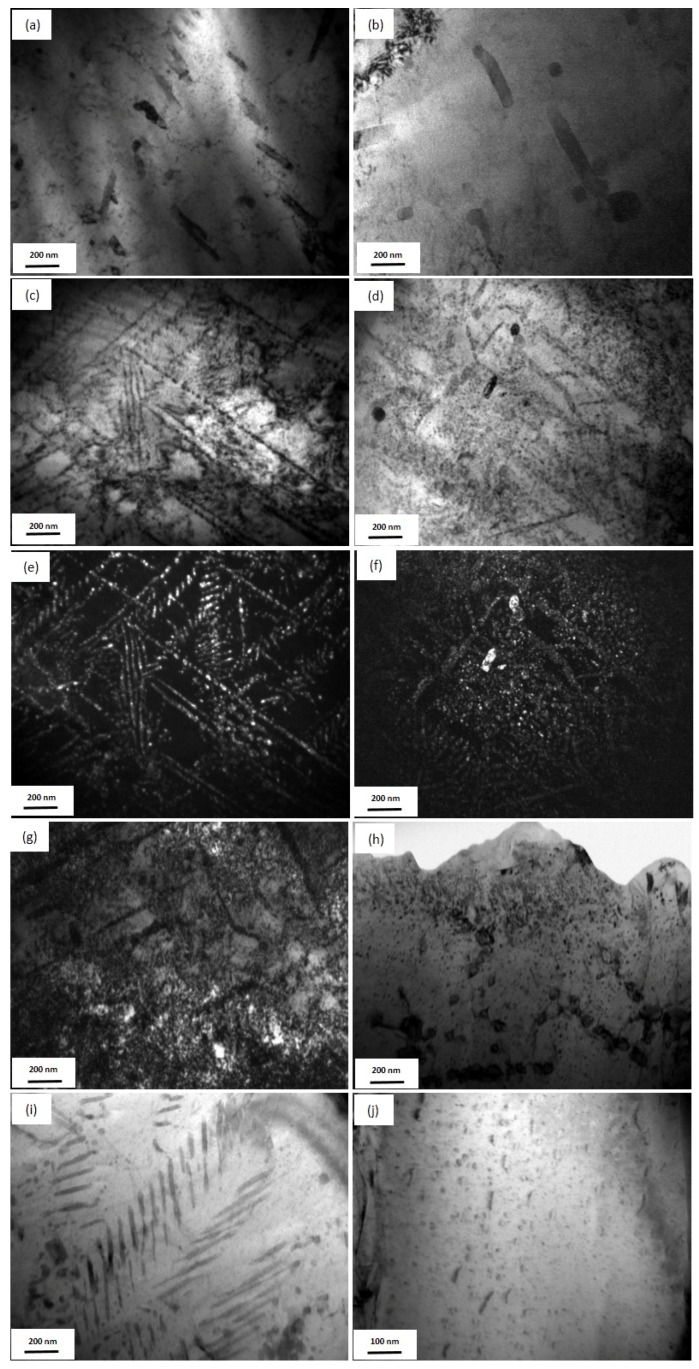
TEM images showing Ag precipitates in the Cu matrix at various temperatures for 4 h. Fe-free alloy: (**a**) as-solid-solution, (**c**) 450 °C and (**e**) dark field image of 450 °C, (**g**) 500 °C, (**i**) 550 °C; Fe-doping alloy: (**b**) as-solid-solution, (**d**) 450 °C and (**f**) dark field image of 450 °C, (**h**) 500 °C, (**j**) 550 °C.

**Figure 5:**
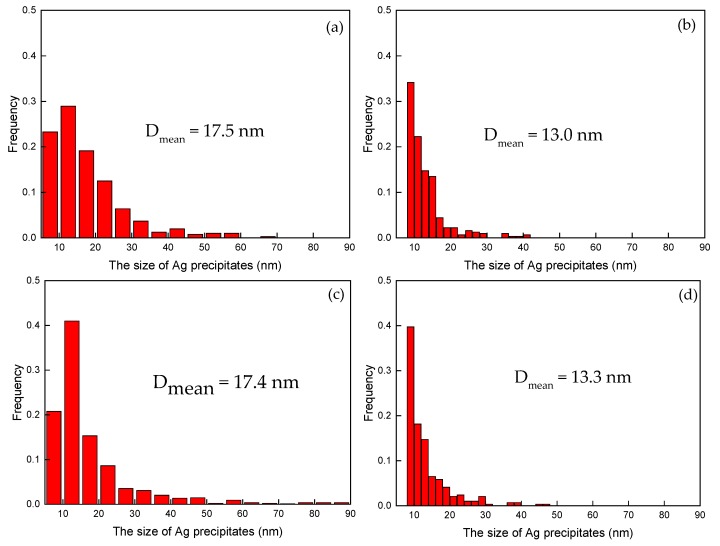
Histogram of the distribution of Ag precipitates in the Cu matrix at various temperatures for 4 h. Fe-free alloy: (**a**) 450 °C; (**c**) 500 °C; Fe-doping alloy: (**b**) 450 °C; (**d**) 500 °C.

The two post-quenched alloys showed small volume fractions of Ag precipitates out of the Cu matrix (Figure 4a,b). The Ag precipitates are needle-like structures with diameters of about 40 nm and lengths of 200 nm. After the Fe-free alloy was aged, the continuous rod-shaped Ag precipitates, with diameters of about 17 nm, were arrayed in parallel, and the spacing was less than 20 nm (Figure 4c,e). Two types of Ag precipitates were found in the Fe-doping alloy aged at 450 °C (Figure 4d,f). The first type was composed of a few large-sized Ag precipitates with diameters of 40 nm; however, the second type was finely distributed in the Cu matrix, and the size was lower than 10 nm. After ageing at 500 °C and 550 °C, the Ag precipitates (Figure 4g,i) in the Fe-free alloys grew with a larger size than the precipitates at 450 °C. In the aged Fe-doping alloys (Figure 4h,j), the diameter and length of continuous rod-shaped Ag particles decreased to about 10 nm and 25 nm, respectively. The spacing decreased to about 20 nm. The size distribution of Ag precipitates (Figure 5a–d) in the Cu matrix of both the Fe-free and Fe-doping alloys were analyzed. The size of Ag precipitates in the Fe-free alloy, with a diameter of ~17.5 nm, was larger than those in the Fe-doping alloy, with a diameter of 13 nm. The size of the Ag precipitates decreased by 23.5% with the Fe addition. In summary, Fe addition decreased both the size and the spacing of the Ag precipitates.

The solute concentration of Ag in the Cu matrix at various temperatures was determined by the EDS in the TEM microstructures of Figure 4. The results are summarized in Table 1. The measured solute concentration of Ag in the Cu matrix in the post-quenched Fe-free alloy is about 3.94 at % (6.49 wt %), which is lower than that of 5.72 at % (9.45 wt %) in the Fe-doping alloy. After ageing treatment, the solute concentration of Ag in the Cu matrix for both the Fe-free and Fe-doping alloys was reduced, because of the precipitation of Ag. In Fe-free aged samples, the Cu matrix had a lower solute concentration of Ag than the matrix in the Fe-doping aged samples. The volume fraction of the Ag precipitates of both of the aged samples was also calculated (shown in Table 1). Ageing at 450 °C, the volume fraction of Ag precipitates evidently increased. As ageing temperature was higher than 450 °C, the volume fraction of Ag precipitates slightly decreased, because of the dissolution of Ag into the Cu matrix. After ageing at 550 °C, the total concentration (both solute concentration and precipitates) of Ag (4.26 wt %) in the Cu matrix in Fe-free alloy was apparently lower than that in the Fe-doping alloy (4.96 wt %). Fe addition increased the solute concentration of Ag in the Cu matrix during ageing. Xie et al. [31] revealed that the presence of Ag inhibits the solubility of Fe in the Cu matrix at a high temperature, because Ag had priority to dissolve in Cu, compared with Fe, by first principle calculations. In other words, Fe precipitates in our experiments were promoted to precipitate out of the Cu matrix because of the presence of more trapped Ag.

#### 3.2.3. Morphology of Fe Precipitates in the Cu Matrix

**Figure 6:**
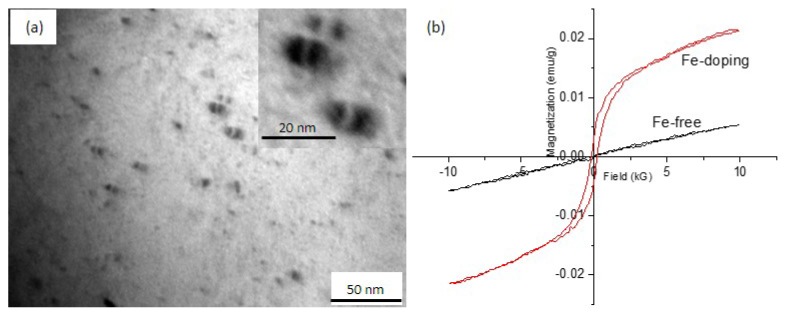
(**a**) TEM bright field image showing γ-Fe precipitates in the Cu matrix of the Fe-doping alloy aged at 550 °C for 4 h, (**b**) Magnetization curves of the Fe-free and Fe-doping alloys aged at 550 °C for 4 h at room temperature. The inset is the high-magnification image showing the γ-Fe precipitates.

Fe precipitates with diameters of ~5 nm were observed after ageing at 550 °C in Fe-doping alloy (Figure 6a). Our previous result indicated that Fe precipitates and the Cu matrix had a cube-on-cube orientation relationship [21]. Magnetization curves of both the Fe-free alloy and the Fe-doping alloy after a heat treatment, aged at 550 °C for 4 h, are shown in Figure 6b. Slight magnetization is observed in the Fe-free alloy. In the Fe-doping sample, a hysteresis loop was observed. The magnetization is 21.53 × 10^−3^ emu/g, and the remanence is 3.8 × 10^−3^ emu/g. The magnetization in the Fe-doping sample might have been the result of the precipitation of γ-Fe precipitates. The solubility of Fe in Cu can be estimated by the magnetization. The saturation magnetization of Fe at room temperature is 274.6 μWbm/kg [45]. If all the Fe atoms in the Fe-doping (0.1 wt %) alloy are precipitated out of the Cu matrix, the magnetization is estimated at 0.2746 μWbm/kg. According to the measured magnetization value of Fe-doping alloy, the solubility of Fe in Cu is determined to be 0.09%. In other words, the fraction of precipitation of γ-Fe out of the Cu matrix is 0.01%.

### 3.3. Hardness and Tensile Strength of Alloys

**Figure 7:**
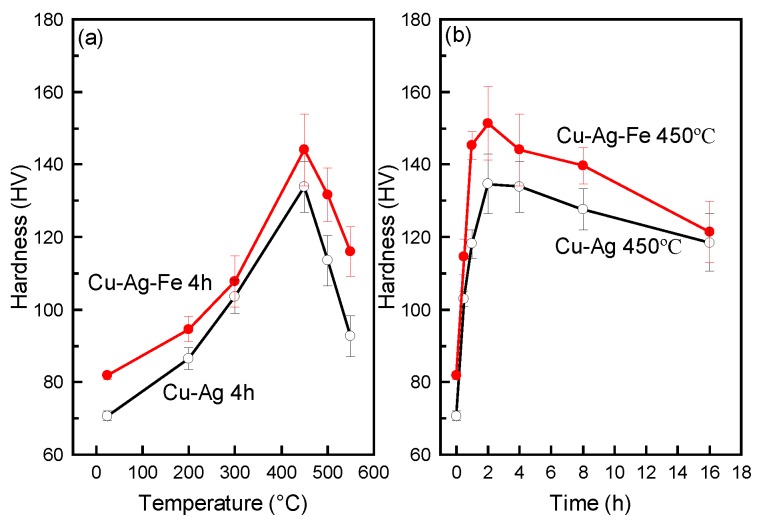
Hardness of aged alloys: (**a**) at different temperatures for 4 h, (**b**) at 450 °C for different times.

As the ageing temperature increased to 450 °C (Figure 7a), the hardness of the Fe-free alloy evidently increased. A maximum value of about 134 HV was observed in the sample after ageing at the temperature of 450 °C, which was an increase of 89.4% compared to that of as-solid-solution sample. The increase of hardness of the Fe-free alloy is likely attributed to the continuous Ag precipitates out of the Cu matrix. The result of DSC (Figure 1) indicated that the range of precipitation temperature of Ag precipitates was between 367 °C and 425 °C. The ageing at this range triggers an Ag precipitation reaction, which leads to a rapidly increasing hardness in the Fe-free alloy. With further increasing of the ageing temperature, the hardness decreases slightly. This is caused by the growth and coarsening of Ag precipitates at high temperatures. With respect to the Fe-doping alloy, a similar tendency is found, and the maximum hardness value is about 144 HV in the sample aged at the temperature of 450 °C, which is a 76.1% increase compared to that of the as-solid-solution sample. The 0.1 wt % Fe addition in the Fe-doping alloy caused increased hardness after ageing at 450 °C by 7.6%. This could be attributed to the precipitation hardening of Fe precipitates, and may reduce the size and spacing of Ag precipitates in the Cu matrix. With ageing temperature over 450 °C, the hardness of both the Fe-free and Fe-doping alloys continued to decrease because of the dissolution of Ag precipitates into the Cu matrix. Ageing at 450 °C for different amounts of time (Figure 7b), the hardness of both alloys increases apparently, and reaches maximum values at an ageing time of 2 h. This indicated that the nucleation and growth of Ag precipitates had a similar tendency in both the Fe-free and Fe-doping alloys.

**Figure 8:**
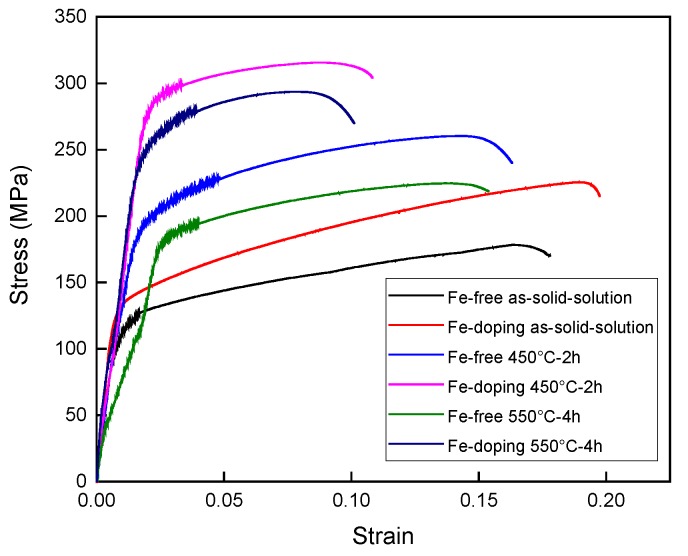
Stress–strain curves of as-solid-solution and ageing-treated, Fe-free and Fe-doping alloys.

Tensile tests for the Fe-free and Fe-doping alloys were carried out at room temperature on as-solid-solution and ageing-treated samples, as shown in Figure 8. The as-solid-solution samples exhibited very low tensile strength, yield strength and elongation, as shown in Table 2. For the Fe-free alloy, the tensile strength and yield strength were 180.4 MPa and 113.6 MPa, respectively. For Fe-doping alloy, the tensile strength and yield strength were 225.4 MPa and 126.1 MPa, respectively. The elongation for both alloys was 13.5% and 13.7%, respectively. Compared with the as-solid-solution samples, the tensile strength, yield strength and elongation of ageing-treated samples all increased after being aged at 450 °C for 2 h. The tensile strength and yield strength were 260.3 MPa and 201.1 MPa for the Fe-free alloy. For the Fe-doping alloys, tensile strength and yield strength were 21.3% and 41.3% higher, respectively, than those of the Fe-free alloy, as also shown in Table 2. With the ageing temperature increasing up to 550 °C, the tensile strength, yield strength and elongation of both alloys decreased. The tensile strength and yield strength of the Fe-free alloy decreased to 224.7 MPa and 187.3 MPa. For the Fe-doping alloy, the tensile strength and yield strength decreased to 293.6 MPa and 252.2 MPa, which is 30.7% and 34.7% higher, respectively, than those of the Fe-free alloy. The apparent decrease of tensile strength and yield strength for the Fe-free alloy was due to the Ag dissolved into the Cu matrix, which weakened the precipitation hardening of Ag precipitates. However, the few decreases of tensile strength and yield strength for the Fe-doping alloy were a result of the precipitation of Fe precipitates out of the Cu matrix, which caused the precipitation hardening.

### 3.4. Electrical Resistivity

**Figure 9:**
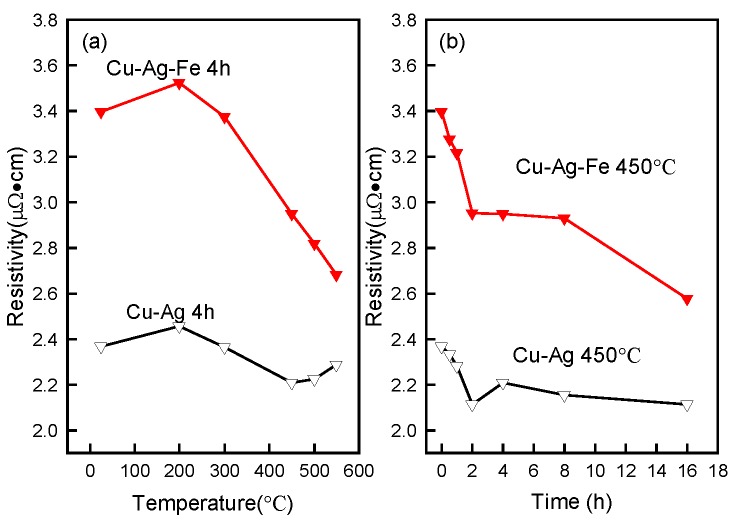
Electrical resistivity of aged alloys: (**a**) at different temperature for 4 h, (**b**) at 450 °C for different time.

Figure 9a shows the influence of the different ageing temperatures on the electrical resistivity for both the Fe-free and Fe-doping alloys. The resistivity of the as-solid-solution Fe-doping alloy was much higher than that of the Fe-free alloy. After ageing at 200 °C, the resistivity of both alloys began to increase slightly. The resistivity began to decrease with ageing temperatures over 200 °C for both alloys. At temperatures above 300 °C, the rate of resistivity appears to drop. The resistivity of the Fe-free alloy reached its lowest value around 450 °C. With ageing temperatures over 450 °C, the resistivity of the Fe-doping alloy continued to decrease, until ageing temperature 550 °C. The decrease of resistivity contributed to the Fe precipitates out of the Cu matrix, reducing the impurity scattering caused by Fe. Compared with the Fe-doping alloy, the resistivity of the Fe-free alloy started to increase slightly. The slight increase of resistivity was due to the dissolution of Ag into the Cu matrix. Figure 9b shows the resistivity of Fe-free and Fe-doping alloys aged at 450 °C as a function of ageing time. The electrical resistivity decreased with ageing time for both alloys. The resistivity of both alloys began to decrease sharply before ageing for 2 h. After prolonging the ageing time to 8 h, the resistivity of both alloys showed almost no change. At ageing time over 8 h, the resistivity of Fe-doping began to decrease rapidly. When the ageing time was 16 h, the electrical resistivity of the Fe-doping alloy was at its minimum, where the electrical conductivity was about 67% IACS (International Annealed Copper Standard). This means that the optimization of heat treatments will increase the electrical conductivity of Cu–Ag–Fe alloys.

## 4. Discussion

### 4.1. The Effect of Fe-Doping on the Precipitation of Ag Precipitates

The morphology of continuous precipitates is determined by the balance between interfacial energy and elastic strain energy during solid–solid transformation. The interfacial energy favors spherical small precipitate particles, while the elastic strain energy favors thin-sheet precipitates [46]. The interfacial energies of the (111) and (001) Cu/Ag interfaces are 0.23 and 0.53 J/m^2^, respectively [47]. Generally, Ag precipitates nucleate and grow along with {111} planes. The elongated shapes of the Ag precipitates are aligned on {111} planes, because of the large anisotropy in the Cu/Ag interfacial energy. In other words, the Ag precipitate adopts a rod shape because its total interfacial energy is smaller than the adjacent respective Ag precipitates, causing strain fields, which bring large strain energy. In addition, the sizes of precipitates are controlled by a soft impingement process, which affects the growth rate [48]. The effect of soft impingement on the overall precipitation reaction is only related to the degree of super-saturation, in the case of the diffusion-controlled growth model [49]. Fe addition reduces the numbers of continuous rod-shaped Ag particles with diameters of <10 nm and lengths in the order of 50 nm (Figure 4j). Because of the precipitation of Fe out of the Cu matrix, the Ag precipitates are adjacent to the Fe precipitates, which can overlap their elastic strain field. Soft impingement refers to the overlapping of diffusion fields around the adjacent growing precipitates, which inhibits the nucleation process. Therefore, the nucleation process of Ag precipitates aligned on {111} planes is inhibited by the overlapping of diffusion fields around the adjacent growing Fe precipitates, so that the size of continuous Ag precipitates is decreased compared to those from the Fe-free alloy.

The solubility limit of Ag in the Cu matrix is related to the size of Ag dispersoids, as expressed by the Gibbs–Thomson formula [50,51]:

(2) $$c_{d}=c_{\propto }\exp(\frac{6\gamma V_{m}}{d\text{R}T})$$

where *d* is the diameter of the initial Ag precipitates, *T* is the temperature of ageing (550 °C), *c_∞_* (0.33 at %) and *c_d_* are the solute Ag concentrations in the Cu matrix, with Ag precipitates having large diameters and diameter *d*, respectively, R is the molar gas constant, *V_m_* is the molar volume fraction of Ag (10^−5^ m^3^/mole), and *ϒ* is the interface energy between the Cu matrix and the Ag precipitates. The value of *ϒ* of the (111) and (001) Cu/Ag interfaces is 0.23 and 0.53 J/m^2^, respectively. According to the Equation (2), by using the value of the diameter of the Ag precipitates (shown in Table 1), the solute concentration of Ag in the Cu matrix of Fe-free and Fe-doping alloys, aged at 550 °C for 4 h, can be calculated to be 0.8 at % and 1.01 at %, respectively. The calculated values of both alloys are much lower than those values measured (shown in Table 1) using EDS. The equilibrium solubility limit of Ag in Cu [52] at the temperature of ageing of 550 °C is about 1.5 at %. The measured value of the solute concentration of Ag in Cu for the Fe-free alloy is 1.64 at %, which is higher than the equilibrium solubility limit (1.5 at %) of Ag in Cu. The higher solute concentration of Ag in the Cu matrix results from the nano-sized Ag precipitates and Fe precipitates precipitated during ageing. Kaptay et al. found that these nanostructured materials could enhance solubilities [53]. The nano-sized Ag precipitates and Fe precipitates caused large interface energy, which raises the free energy, and consequently the equilibrium solubility of Ag in the Cu matrix is enhanced.

### 4.2. The Effect of Fe-Doping on Strength

Both of ageing-treated Fe-free and Fe-doping alloys consisted of two components: a proeutectic Cu (Cu matrix) and a eutectic. The contribution of the two components to the strength *τ* can be described by the rule of mixture [10], as shown in Equation (3);

(3) $$\tau =(1-V_{\mathrm{eut}}{)\tau }_{\mathrm{Cu}\;\mathrm{matrix}}{+V}_{\mathrm{eut}}{(\tau }_{\mathrm{eut}})$$

where *V*_eut_ is the volume fraction of the eutectic, *τ*_Cu matrix_ is the strength of the Cu matrix, and *τ*_eut_ is the strength of the eutectic.

The strength of the eutectic (*τ*_eut_) was determined by the pure Ag. The strength of pure Ag is 37 MPa [30]. The volume fraction of the eutectic in the Fe-free and Fe-doping alloys, after being aged at 550 °C for 4 h, was 25.0% and 26.2%, respectively. Consequently, the strength of the eutectic was calculated to be 9.25 MPa and 9.7 MPa for the Fe-free and Fe-doping alloys, respectively.

The strength of the Cu matrix (*τ*_Cu matrix_) was determined by the superposition of several strengthening partitions: solid solution hardening (*τ*_ss_), precipitation hardening (*τ*_Precipitation_), and grain boundary hardening (*τ*_Grain_) [54]. The strength of the Cu matrix increased due to these individual contributions, which can be formulated by Equation (4):

(4) $$\tau_{\mathrm{Cu}\;\mathrm{matrix}\;}{=\;\tau }_{\mathrm{SS},\mathrm{Ag}}{\;+\;\tau }_{\mathrm{SS},\mathrm{Fe}}\;+\;\tau_{\mathrm{Pre},\mathrm{Ag}}{\;+\;\tau }_{\mathrm{Pre}\;\mathrm{Fe}}\;+\;\tau_{\mathrm{Gb}}$$

where *τ*_ss,Ag_ and *τ*_ss,Fe_ are the solid solution hardening of supersaturated Ag and Fe in the Cu matrix. *τ*_Pre,Ag_ and *τ*_Pre,Fe_ are the precipitation hardening of Ag precipitates and Fe precipitates out of the Cu matrix.

Solid solution hardening (*τ*_ss_) can be calculated using Equation (5) [54,55]:

(5) $$\tau_{\mathrm{ss}}=G{\left(\left|\delta \right|+\frac{1}{20}\left|\eta \right|\right)}^{3/2}\sqrt{\frac{\chi_{a}}{3}}$$

where *G* is the shear modulus of the alloy, estimated according to the rules of mixture, *δ* is a factor of lattice change, *η* is the change of the shear modulus of alloying, and *χ_a_* is the atomic fraction within the solid solution. In the Fe-doping alloy, the solid solution hardening includes both supersaturated Ag and Fe in the Cu matrix. Using the measured results of solute concentration of Ag and Fe in the Cu matrix, the solid solution hardening of supersaturated Ag and Fe was estimated. The *χ_a,_*_Ag_ was measured to be 1.16 at % and 1.64 at % for as-solid-solution Fe-free and Fe-doping alloys, respectively. The strength values resulting from the solid solution hardening of Ag were calculated to be 147.0 MPa and 176.1 MPa respectively for Fe-free and Fe-doping alloys aged at 550 °C. The *χ_a_*_,Fe_ was estimated at 0.14 at % for the Fe-doping alloy. The strength value resulting from the solid solution hardening of Fe was calculated as 52.1 MPa for the Fe-doping alloy aged at 550 °C. This indicated that solid solution hardening of Fe also plays a major role in the hardening of the Fe-doping alloy.

Precipitation hardening (*τ*_Pre, Ag_) can be estimated by the following Orowan–Ashby Equation (6) [55]:

(6) $${\Delta \tau }_{P}=\frac{Gb\sqrt{f}}{r}$$

where ***b*** is the Burgers vector (0.2556 nm for Cu), *r* is the particle radius, and *f* is volume fraction of Ag and Fe precipitates.

The *r* of Ag precipitates in 550 °C, 4 h-aged Fe-free and Fe-doping samples was 8.8 nm and 4.1 nm, respectively; the *f* for both samples was 2.3% and 2.2% (shown in Table 1). Thus, precipitation hardening of the Ag precipitates was calculated as 184 MPa and 393 MPa respectively for Fe-free and Fe-doping samples aged at 550 °C for 4 h.

The *r* of the Fe precipitates in 550 °C-4 h-aged Fe-doping samples was about 3.5 nm, and the volume fraction was estimated at 0.01%. Thus, precipitation hardening of Fe precipitates was calculated as 31.1 MPa for the Fe-doping alloy aged at 550 °C for 4 h.

We neglected the grain boundary hardening because of the large grain size in aged samples.

The total strength of the Cu matrix in both alloys was calculated to be 340.3 MPa and 662.0 MPa.

According to Equation (3), the total tensile strength of both alloys aged at 550 °C for 4 h was estimated at 340.3 MPa and 662.0 MPa, respectively, which deviated from the measured values of tensile strength. This might be attributed to the higher calculated precipitation hardening for both alloys. The Orowan–Ashby Equation (6) is based on the large spacing between two particle precipitates, where the spacing is far larger than the diameter of particle precipitates. In our results, the spacing of both alloys was not much larger than the diameters of precipitates. Therefore, the calculated values of precipitation hardening from precipitates are a bit high.

The individual contributions to the strength for both alloys summarized in Table 3 show that Ag precipitates play an important role in strengthening alloys. In addition, the presence of Fe also plays a dominant role in strengthening alloys. On the one hand, Fe promotes the dissolving of Ag into the Cu matrix, which enhances solid solution hardening; on the other hand, Fe precipitation out of the Cu matrix also causes precipitation hardening, especially when Fe precipitates can be precipitated much more from the Cu matrix.

### 4.3. The Effect of Fe-Doping on Electrical Resistivity

The resistivity of the aged Fe-free and Fe-doping alloys can be described by the contributions of four scattering mechanisms [3]:

(7) $${\rho =\rho }_{\mathrm{pho}}{+\rho }_{\mathrm{dis}}{+\rho }_{\mathrm{int}}{+\rho }_{\mathrm{imp}}$$

where *ρ*_pho_ is the resistivity contribution from phonon scattering, *ρ*_dis_ is dislocation scattering, *ρ*_int_ is the interface scattering, and *ρ*_imp_ is the impurity scattering, depending on the rules of mixture. For this equation, *ρ*_Fe-free, pho_ = 1.64 μΩ·cm is determined upon the volume fraction of Cu (x_Cu_ = 0.734) and Ag (x_Ag_ = 0.266) on the basis of the value for pure Cu (*ρ*_Cu_ = 1.667 μΩ·cm) and Ag (*ρ*_Ag_ = 1.559 μΩ·cm) [30]. *ρ*_Fe-doping, pho_ = 1.67 μΩ·cm is estimated from the volume fraction of Cu (x_Cu_ = 0.780), Ag (*x*_Ag_ = 0.219) and Fe (*x*_Fe_ = 0.001) on the basis of the value for pure Cu (*ρ*_Cu_ = 1.667 μΩ·cm), Ag (*ρ*_Ag_ = 1.559 μΩ·cm) and Fe (*ρ*_Fe_ = 9.78 μΩ·cm). Gaganov et al. [19] found that *ρ*_dis_ = 0.000075 μΩ·cm without any deformation (the dislocation density is *N* = 5 × 10^9^ m^−2^). Interface scattering in both aged alloys includes the grain and phase boundaries of both precipitates and eutectics. Resistivity is due to impurity scattering results from the supersaturated Ag and Fe in the Cu matrix. The resistivity increase per 1 at % Ag addition is 2.63 nΩ·m [30]. The resistivity increase per 1 wt % Fe addition is 9.2 μΩ·cm [28]. For the 550 °C-4 h-aged Fe-free alloy, the solubility of Ag in Cu was 1.64 at %. So *ρ*_Fe-free imp_ is estimated at 0.0305 μΩ·cm. For the 550 °C-4 h-aged Fe-doping alloy, the solubility of Ag and Fe in Cu was 1.16 at % and 0.09 wt %, respectively. In addition, *ρ*_Fe-doping imp_ was determined to be 0.9178 μΩ·cm.

The contributions from individual portions’ resistivity are summed up here. For the 550 °C-4 h aged Fe-free alloy, a *ρ*_Fe-free_ = 1.671 μΩ·cm is found. For the Fe-doping alloy, *ρ*_Fe-doping_ = 2.588 μΩ·cm (shown in Table 4). Both calculated values are slightly lower than that of measured values from experiments. The deviations are likely caused by the interface scattering resistivity, which is exclusive to our work.

The individual contributions to the resistivity for both alloys, summarized in Table 4, shows that the impurity scattering caused by supersaturated Fe is the main reason for the high resistivity of the Fe-doping alloy.

### 4.4. The Diagram between Electrical Conductivity and Strength

**Figure 10:**
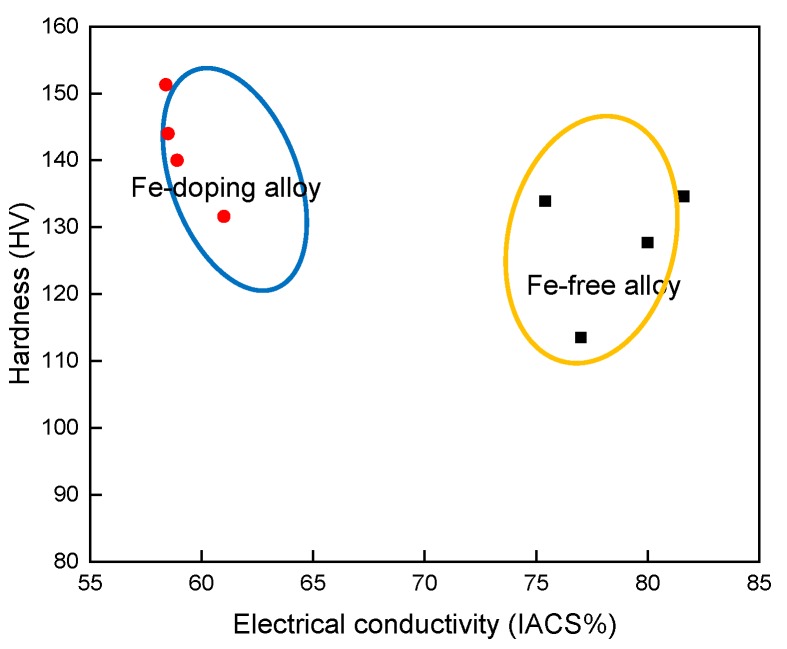
The relationship of hardness and electrical conductivity of Fe-free and Fe-doping alloys.

The relationship between the hardness and electrical conductivity of the two alloys after various ageing treatments (Figure 10) shows that the Fe-doping alloys kept a lower level of electrical conductivity with 60% IACS compared with the Fe-free alloy; however, the hardness of the alloy with the Fe-addition increased noticeably. The maximum hardness value of the Fe-doping alloy can be reached at 151 HV, which is12.7% greater than the maximum of the Fe-free alloy. These data show that Cu–Ag–Fe alloy has a potential application in transmission lines.

## 5. Conclusions

(1) In a Cu–26 wt % Ag alloy, the activation energy of Ag precipitated out of the Cu matrix was 66.4 ± 6.4 kJ/mol, and the activation energy of Cu precipitated out of the Ag matrix was 125 ± 13.8 kJ/mol. With respect to the Cu–26 wt % Ag–0.1 wt % Fe alloy, these two energies were 63.5 ± 3.2 kJ/mol and 129 ± 8.9 kJ/mol, respectively. Fe-doping had a negligible influence on the activation energies of Ag and Cu precipitates.
(2) The continuous rod-shaped Ag precipitates precipitated out of the Cu matrix of both the Fe-free alloy as well as the Fe-doping alloy after ageing treatments. The Fe addition decreased the size and the spacing of the continuous rod-shaped Ag precipitates, because the elastic strain field caused by the presence of the overlapped Fe diffusion field inhibited the nucleation of Ag precipitates.
(3) The electrical resistivity of the Fe-free alloy slightly increased after ageing at 550 °C, due to the dissolution of Ag into the Cu matrix. Fe-doping alloy aged at 550 °C-4 h indicated that γ-Fe did precipitate from the Cu matrix, and that the solubility of Fe in Cu was about 0.09%. Thus, the electrical resistivity of Fe-doping alloy decreased after ageing.

The strength and hardness of the Fe-doping alloy were higher than those of the Fe-free alloy. The uniformly dispersed nano-sized Ag and Fe precipitates were co-precipitated after heat treatments, resulting in the precipitation hardening.

## Figures and Tables

**Table 1:** Microstructural characterization of Fe-free and Fe-doping alloys at various temperatures.

Alloy	Temperature	Mean Diameter *d* (nm)	Spacing *λ* (nm)	Measured Solute Ag Concentration in Cu, *C_M_._Ag_* (at %, wt %)	Measured Volume Fraction of Ag Out of Cu, *V_M_._f_* (%)
Fe-free	As-solid-solution	--	--	3.94 (6.49)	3.2
	450 °C-4 h	17.5	35.7 ± 1.2	3.0 (4.98)	4.2
	500 °C-4 h	17.4	15.8 ± 1.4	4.53 (7.46)	2.7
	550 °C-4 h	17.6	29.6 ± 1.4	1.16 (1.96)	2.3
Fe-doping	As-solid-solution	--	--	5.72 (9.45)	1.4
	450 °C-4 h	13.0	18.2 ± 0.2	5.08 (8.32)	2.7
	500 °C-4 h	13.3	15.7 ± 1.4	4.83 (7.93)	2.6
	550 °C-4 h	8.2	17.5 ± 1.5	1.64 (2.76)	2.2

**Table 2:** The strength and elongation of Fe-free and Fe-doping alloys.

		Tensile Strength (MPa)	Yield Strength (MPa)	Elongation (%)
Fe-free alloy	As-solid-solution	180.4	113.6	13.5
	450 °C-2 h	260.3	201.1	15.4
	550 °C-4 h	224.7	187.3	14.2
Fe-doping alloy	As-solid-solution	225.4	126.1	13.7
	450 °C-2 h	315.8	284.2	14.2
	550 °C-4 h	293.6	252.2	13.9

**Table 3:** Individual contributions to the strength of both Fe-free and Fe-doping alloys aged at 550 °C for 4 h.

		Fe-Free		Fe-Doping
Strength of the Cu matrix *τ*_Cu matrix_ (MPa)	Solid solution hardening *τ*_ss_	Ag	147.0	176.1
		Fe	--	52.1
	precipitation hardening *τ*_Pre_	Ag	184	393
		Fe	--	31.1
Strength of eutectic *τ*_eut_ (MPa)			9.3	9.7
Total strength *τ*_total_ (MPa)			340.3	662.0
Measured strength *τ*_measured_ (MPa)			224	293

**Table 4:** Individual contributions to the resistivity of both Fe-free and Fe-doping alloys aged at 550 °C for 4 h.

	Phonon Scattering *ρ*_pho_ μΩ·cm	Dislocation Scattering *ρ*_dis_ μΩ·cm	Impurity Scattering *ρ*_imp_ μΩ·cm		Total Resistivity *ρ*_total_ μΩ·cm	Measured Resistivity *ρ*_measured_ μΩ·cm
Fe-free alloy	1.64	0.000075	Ag	0.0305	1.671	2.2875
			Fe	--		
Fe-doping alloy	1.67	0.000075	Ag	0.043	2.588	2.6801
			Fe	0.8748		

## References

[1] Embury J.D., Han K. (1998). Conductor materials for high field magnets. Curr. Opin. Solid State Mater. Sci..

[2] Sakai Y., Inoue K., Maeda H. (1995). New High-Strength, High-Conductivity Cu-Ag Alloy Sheets. Acta Metall. Mater..

[3] Hong S.I., Hill M.A. (1999). Mechanical stability and electrical conductivity of Cu-Ag filamentary microcomposites. Mater. Sci. Eng. A.

[4] Verhoeven J., Downing H., Chumbley L.S., Gibson E. (1989). The resistivity and microstructure of heavily drawn Cu-Nb alloys. J. Appl. Phys..

[5] Biselli C., Morris D.G. (1996). Microstructure and strength of Cu-Fe in situ composites after very high drawing strains. Acta Mater..

[6] Islamgaliev R.K., Nesterov K.M., Valiev R.Z. (2015). Structure, strength, and electric conductivity of a Cu-Cr copper-based alloy subjected to severe plastic deformation. Phys. Met. Metallogr..

[7] Sakai Y., Inoue K., Maeda H. (1994). High-strength and high-conductivity Cu-Ag alloy sheets: New promising conductor for high-fieId Bitter coils. IEEE Trans. Magn..

[8] Zuo X.W., Zhao C.C., Niu R.M., Wang E.G., Han K. (2015). Microstructural dependence of magnetoresistance in CuAg alloy solidified with high magnetic field. J. Mater. Process. Technol..

[9] Benghalem A., Morris D.G. (1997). Microstructure and strength of wire-drawn Cu-Ag filamentary composites. Acta Mater..

[10] Han K., Vasquez A.A., Xin Y., Kalu P.N. (2003). Microstructure and tensile properties of nanostructured Cu-25 wt % Ag. Acta Mater..

[11] Liu J.B., Meng L., Zeng Y.W. (2006). Microstructure evolution and properties of Cu-Ag microcomposites with different Ag content. Mater. Sci. Eng. A.

[12] Zuo X.W., Han K., Zhao C.C., Niu R.M., Wang E.G. (2014). Microstructure and properties of nanostructured Cu-28 wt % Ag microcomposite deformed after solidifying under a high magnetic field. Mater. Sci. Eng. A.

[13] Zhao C., Zuo X., Wang E., Niu R., Han K. (2016). Simultaneously increasing strength and electrical conductivity in nanostructured Cu-Ag composite. Mater. Sci. Eng. A.

[14] Zuo X.W., Han K., Zhao C.C., Niu R.M., Wang E.G. (2015). Precipitation and dissolution of Ag in ageing hypoeutectic alloys. J. Alloys Compd..

[15] Zhao C.C., Zuo X.W., Wang E.G., Han K. (2017). Strength of Cu-28 wt % Ag Composite Solidified Under High Magnetic Field Followed by Cold Drawing. Met. Mater. Int..

[16] Zuo X.W., Guo R., Zhao C.C., Zhang L., Wang E.G., Han K. (2016). Microstructure and properties of Cu-6 wt % Ag composite thermomechanical-processed after directionally solidifying with magnetic field. J. Alloys Compd..

[17] Nestorovic S., Markovic I., Markovic D. (2010). Influence of thermomechanical treatment on the hardening mechanisms and structural changes of a cast Cu-6.6 wt % Ag alloy. Mater. Des..

[18] Piyawit W., Xu W.Z., Mathaudhu S.N., Freudenberger J., Rigsbee J.M., Zhu Y.T. (2014). Nucleation and growth mechanism of Ag precipitates in a CuAgZr alloy. Mater. Sci. Eng. A.

[19] Gaganov A., Freudenberger J., Botcharova E., Schultz L. (2006). Effect of Zr additions on the microstructure, and the mechanical and electrical properties of Cu-7 wt % Ag alloys. Mater. Sci. Eng. A.

[20] Raabe D., Hangen U. (1996). Correlation of microstructure and type II superconductivity of a heavily cold rolled Cu-20 mass % Nb in situ composite. Acta Mater..

[21] Zuo X.W., Qu L., Zhao C.C., An B.L., Wang E.G., Niu R.M., Xin Y., Lu J., Han K. (2016). Nucleation and growth of gamma-Fe precipitate in Cu-2% Fe alloy aged under high magnetic field. J. Alloys Compd..

[22] Wu Z.W., Chen Y., Meng L. (2009). Microstructure and properties of Cu-Fe microcomposites with prior homogenizing treatments. J. Alloys Compd..

[23] Wu Z.W., Meng L. (2011). Influences of different prior heat treatments on the microstructural and mechanical properties of Cu-Fe filamentary composites. J. Alloys Compd..

[24] Davis J.R. (1992). ASM Handbook Volume 3: Alloy Phase Diagrams.

[25] Easterling K.E., Weatherly G.C. (1969). On the nucleation of martensite in iron precipitates. Acta Metall..

[26] Saji S., Hori S., Mima G. (1973). Ageing Characteristics of Copper-Iron Alloys. Mater. Trans. JIM.

[27] Nakagawa Y. (1958). Liquid immiscibility in copper-iron and copper-cobalt systems in the supercooled state. Acta Metall..

[28] Verhoeven J., Chueh S., Gibson E. (1989). Strength and conductivity ofin situ Cu-Fe alloys. J. Mater. Sci..

[29] Go Y., Spitzig W. (1991). Strengthening in deformation-processed Cu-20% Fe composites. J. Mater. Sci..

[30] Smith D.R., Fickett F. (1995). Low-temperature properties of silver. J. Res. Natl. Inst. Stand. Technol..

[31] Xie Z., Gao H.Y., Lu Q., Wang J., Sun B.D. (2010). Effect of Ag addition on the as-cast microstructure of Cu-8 wt % Fe in situ composites. J. Alloys Compd..

[32] Song J.S., Ahn J.H., Kim H.S., Hong S.I. (2001). Comparison of microstructure and strength in wire-drawn and rolled Cu-9 Fe-1.2 Ag filamentary microcomposite. J. Mater. Sci..

[33] Wang Y.F., Gao H.Y., Wang J., Han Y.F., Dai Y.B., Sun B.D. (2014). First-principles calculations of Ag addition on the diffusion mechanisms of Cu-Fe alloys. Solid State Commun..

[34] Liu K.M., Lu D.P., Zhou H.T., Chen Z.B., Atrens A., Lu L. (2013). Influence of a high magnetic field on the microstructure and properties of a Cu-Fe-Ag in situ composite. Mater. Sci. Eng. A.

[35] Gao H.Y., Wang J., Shu D., Sun B.D. (2006). Effect of Ag on the aging characteristics of Cu-Fe in situ composites. Scr. Mater..

[36] Sun B.D., Gao H.Y., Wang J., Shu D. (2007). Strength of deformation processed Cu-Fe-Ag in situ composites. Mater. Lett..

[37] Song J.S., Hong S.I., Kim H.S. (2001). Heavily drawn Cu-Fe-Ag and Cu-Fe-Cu microcomposites. J. Mater. Process. Technol..

[38] Gayler M., Carrington W. (1947). Metallographic study of the precipitation of copper from a silver-rich silver-copper alloy. J. Inst. Met..

[39] Li R., Zuo X.W., Wang E.G. (2017). Microstructure, resistivity, and hardness of aged Ag-7 wt % Cu alloy. Acta Phys. Sin..

[40] Kissinger H.E. (1957). Reaction kinetics in differential thermal analysis. Anal. Chem..

[41] Hamana D., Hachouf M., Boumaza L., Biskri Z.E.A. (2011). Precipitation Kinetics and Mechanism in Cu-7 wt % Ag Alloy. Mater. Sci. Appl..

[42] Colombo S., Battaini P., Airoldi G. (2007). Precipitation kinetics in Ag-7.5 wt %Cu alloy studied by isothermal DSC and electrical-resistance measurements. J. Alloys Compd..

[43] Hamana D., Boumaza L. (2009). Precipitation mechanism in Ag-8 wt %Cu alloy. J. Alloys Compd..

[44] Bizjak M., Kosec L., Kosec B., Anzel I. (2006). The characterization of phase transformations in rapidly solidified Al-Fe and Cu-Fe alloys through measurements of the electrical resistance and DSC. Metalurgija.

[45] Watanabe Y., Murakami J.-I., Miura H. (2002). Effect of annealing on saturation magnetization in deformed Cu-Fe alloys with transformed Fe particles. Mater. Sci. Eng. A.

[46] Nabarro F.R.N. (1940). The Strains Produced by Precipitation in Alloys. Proc. R. Soc. A.

[47] Bacher P., Wynblatt P., Foiles S.M. (1991). A Monte Carlo study of the structur and composition of (001) semicoherent interphase boundaries in Cu Ag Au alloys. Acta Metall. Mater..

[48] Tomellini M. (2011). Impact of soft impingement on the kinetics of diffusion-controlled growth of immiscible alloys. Comput. Mater. Sci..

[49] Hao C., Zwaag S.V.D. (2011). Modeling of soft impingement effect during solid-state partitioning phase transformations in binary alloys. J. Mater. Sci..

[50] Perez M. (2005). Gibbs-Thomson effects in phase transformations. Scr. Mater..

[51] Gubicza J., Hegedűs Z., Lábár J.L., Kauffmann A., Freudenberger J., Subramanya Sarma V. (2015). Solute redistribution during annealing of a cold rolled Cu-Ag alloy. J. Alloys Compd..

[52] Subramanian P., Perepezko J. (1993). The Ag-Cu (silver-copper) system. J. Phase Equilib..

[53] Kaptay G. (2012). Nano-Calphad: extension of the Calphad method to systems with nano-phases and complexions. J. Mater. Sci..

[54] Freudenberger J., Lyubimova J., Gaganov A., Witte H., Hickman A.L., Jones H., Nganbe M. (2010). Non-destructive pulsed field CuAg-solenoids. Mater. Sci. Eng. A.

[55] Gottstein G. (2007). Physikalische Grundlagen der Materialkunde.

